# Biosynthesis, Characterization, and Bioactivities Evaluation of Silver and Gold Nanoparticles Mediated by the Roots of Chinese Herbal *Angelica pubescens* Maxim

**DOI:** 10.1186/s11671-017-1833-2

**Published:** 2017-01-17

**Authors:** Josua Markus, Dandan Wang, Yeon-Ju Kim, Sungeun Ahn, Ramya Mathiyalagan, Chao Wang, Deok Chun Yang

**Affiliations:** 1 Graduate School of Biotechnology and Ginseng Bank, College of Life Science, Kyung Hee University, Yongin-si, Gyeonggi-do 446-701 Republic of Korea; 2 Department of Oriental Medicinal Biotechnology, College of Life Science, Kyung Hee University, Yongin-si, Gyeonggi-do 446-701 Republic of Korea

**Keywords:** *Angelica pubescens* Maxim, Gold nanoparticles, Silver nanoparticles, Antioxidant activity, Antimicrobial activity, Cytotoxicity, RAW264.7

## Abstract

**Abstract:**

A facile synthesis and biological applications of silver (DH-AgNps) and gold nanoparticles (DH-AuNps) mediated by the aqueous extract of Angelicae Pubescentis Radix (Du Huo) are explored. Du Huo is a medicinal root belonging to *Angelica pubescens* Maxim which possesses anti-inflammatory, analgesic, and antioxidant properties. The absorption spectra of nanoparticles in varying root extract and metal ion concentration, pH, reaction temperatures, and time were recorded by ultraviolet–visible (UV-Vis) spectroscopy. The presence of DH-AgNps and DH-AuNps was confirmed from the surface plasmon resonance intensified at ~414 and ~540 nm, respectively. Field emission transmission electron micrograph (FE-TEM) analysis revealed the formation of quasi-spherical DH-AgNps and spherical icosahedral DH-AuNps. These novel DH-AgNps and DH-AuNps maintained an average crystallite size of 12.48 and 7.44 nm, respectively. The biosynthesized DH-AgNps and DH-AuNps exhibited antioxidant activity against 2,2-diphenyl-1-picrylhydrzyl (DPPH) radicals and the former exhibited antimicrobial activity against clinical pathogens including *Escherichia coli*, *Staphylococcus aureus*, *Pseudomonas aeruginosa*, and *Salmonella enterica*. The expected presence of flavonoids, sesquiterpenes, and phenols on the nanoparticle surface were conjectured to grant protection against aggregation and free radical scavenging activity. DH-AgNps and DH-AuNps were further investigated for their cytotoxic properties in RAW264.7 macrophages for their potential application as drug carriers to sites of inflammation. In conclusion, this green synthesis is favorable for the advancement of plant mediated nano-carriers in drug delivery systems, cancer diagnostic, and medical imaging.

**Graphical abstract:**

Schematic diagram of biosynthesis of DH-AgNps and DH-AuNps and evaluation of their bioactivities.
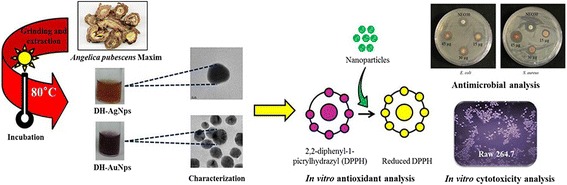

## Background

Nanobiotechnology is a multidisciplinary field that promotes biological constituents with physicochemical processes to manufacture nanomaterials with multifunctional properties. Presently, there is an emerging trend of utilizing biological sources as non-toxic and eco-friendly strategies to develop inorganic nanoparticles with pervasive biomedical applications [1–3]. In addition to avoiding harsh chemicals, medicinal plant-mediated synthesis is preferred due to its rapid, straightforward reaction and augmentation of bioactive phytonutrients that may render nanoparticles biologically active to human applications [4]. Plasmonic metal nanoparticles, such as silver (AgNps) and gold (AuNps), are excellent biosensors and drug delivery agents owing to their remarkable optical and surface functionalization properties [5]. Until now, AgNps and AuNps have been synthesized by various medicinal plant extracts such as *Panax ginseng* Meyer [6–8], *Dendropanax mobifera* Léveillé [9], *Tamarix gallica* [10], and *Terminalia chebula* [11].

Considering the importance of an eco-friendly method as a substitute for chemical reduction and increasing demand for biologically active metal nanoparticles by green chemistry, we reported for the first time a rapid and green synthesis of silver (DH-AgNps) and gold nanoparticles (DH-AuNps) facilitated by Angelicae Pubescentis Radix, otherwise known as the roots of *Angelica pubescens* Maxim. f. *biserrata* Shan et Yuan [12]. For centuries, the roots of *A. pubescens* (a.k.a. Du Huo) have been widely used in traditional Chinese medicine as an alternative treatment of arthritic diseases [13]. The main bioactive component of this herbal drug is coumarins; more than 60 coumarins have been isolated from Angelicae Pubescentis Radix and reported to afford diverse pharmacological activities, such as anti-inflammatory, analgesic, anticancer, and antioxidant [12, 14–16].

The biosynthesized nanoparticles were extensively characterized by spectroscopic and analytical instruments, such as ultra-violet visible (UV-Vis) spectroscopy, field emission transmission electron microscopy (FE-TEM), energy-dispersive X-ray (EDX) spectroscopy, elemental mapping, X-ray powder diffraction (XRD), selected area electron diffraction (SAED), dynamic light scattering (DLS), and Fourier Transform Infrared (FTIR) spectroscopy. For the first time, the effects of root extract and metal ion concentration, pH, reaction temperatures, and time on the biosynthesis of DH-AgNps and DH-AuNps were elucidated. Moreover, *in vitro* antioxidant activity of *A. pubescens* functionalized DH-AgNps and DH-AuNps by 2,2-diphenyl-1-picrylhydrzyl (DPPH) free radical scavenging method was assessed. In addition, DH-AgNps and DH-AuNps were tested for their ability to inhibit the growth of pathogenic microorganisms including *Escherichia coli*, *Staphylococcus aureus*, *Pseudomonas aeruginosa*, and *Salmonella enterica*. Lastly, in vitro cytotoxicity assay of DH-AuNps and DH-AgNps in murine macrophage (RAW264.7) and lipopolysaccharide (LPS)-induced RAW264.7 was examined to explore their potential as novel anti-inflammatory agents.

## Methods

### Materials

Angelicae Pubescentis Radix was procured from Ginseng Bank, Kyung Hee University, Republic of Korea. Silver nitrate (silver salt), hydrogen tetrachloroaurate (III) hydrate (gold salt), DPPH, ascorbic acid (vitamin C), and LPS were purchased from Sigma-Aldrich Chemicals, USA. All other chemicals were of analytical grade and used as received. The pathogenic bacterial strains *E. coli* [ATCC 10798], *S. aureus* [ATCC 6538], *P. aeruginosa* [ATCC 27853], and *S. enterica* [ATCC 13076] were utilized. Standard Neomycin (NEO30) discs were obtained from Oxoid Ltd., England. The bacterial strains were cultured on nutrient agar media at 37 °C for 24 h and preserved at 70 °C in glycerol stock vials for further study.

### Preparation of *Angelicae Pubescentis* Radix Extract

The roots of *A. pubescens* Maxim were washed with distilled water repeatedly to remove impurities. The washed roots were pulverized into fine powder. Next, 5 g of root powder was thoroughly suspended in 100 mL distilled water and autoclaved for 30 min at 100 °C to obtain aqueous root extract. After boiling, the extract was filtered with Whatman grade no. 1 filter paper. The percolated extract was centrifuged at 6300×g for 10 min to remove undesirable solids and the supernatant was collected. The extract was maintained at 100 mL and stored at 4 °C for further use.

### Biosynthesis of DH-AgNps and DH-AuNps

For the synthesis of DH-AgNps and DH-AuNps, silver nitrate and hydrogen tetrachloroaurate (III) hydrate (1, 3, 5, 7, and 9 mM) were dissolved in an aqueous solution containing of Angelicae Pubescentis Radix extract (10, 30, 50, 70, and 90%, v/v). The reaction mixtures were heated at the desired temperature (40, 60, 70, 80 90, and 100 °C) to yield metal nanoparticles. The reaction mixtures were adjusted to different pH values (pH 2, 4, 6, 8, and 12) by drop-wise addition of 0.1 M HCl or 0.2 M NaOH. The characterization sample was obtained at a salt concentration of 5 mM, a root extract concentration of 50% (v/v), a reaction temperature of 80 °C, and a reaction time of 50 and 10 min for DH-AgNps and DH-AuNps, respectively. Color change, due to surface plasmon resonance (SPR), was observed visually, indicating the formation of nanoparticles. Purification and characterization of biosynthesized nanoparticles were performed by a method introduced by Singh et al. with slight alterations [8]. After color change was observed, the reaction mixtures were first centrifuged at 6300×g for 15 min to remove the unreacted plant extract. DH-AgNps and DH-AuNps were further purified by repeated centrifugation at 28,000×g for 10 min at room temperature followed by resuspension in sterile distilled water; this process was carried out repetitively to ensure the removal of unwanted substances. The purified nanoparticles were finally suspended in distilled water and stored at 4 °C in a dark condition. For XRD and FTIR analysis, the purified nanoparticles were air-dried overnight and obtained in powder form.

### Characterization of DH-AgNps and DH-AuNps

The bio-reduction of metal ions into metallic nanoparticles can be verified by monitoring the absorption spectra of the aliquots of the reaction mixtures. The samples were scanned in the range of 300–800 nm by UV-Vis spectrophotometer (Ultrospec^TM^ 2100 pro) with a 10 mm path length quartz cuvette (2100 Pro, Amersham, Biosciences Corp. USA).

The morphology, purity, and elemental distribution of the nanoparticles were assessed by FE-TEM, EDX, SAED, and elemental mapping analysis with a JEM-2100 F (JEOL) instrument operated at 200 kV (JEOL JEM-2100 F, USA). Samples were prepared by placing droplets of the purified nanoparticle suspension onto a carbon-coated copper grid and drying in an oven at 60 °C before transferring to FE-TEM.

Crystallinity of the biosynthesized nanoparticles was examined by a compact XRD instrument (D8 Advance, Bruker, Germany) operating at a voltage of 40 kV and 40 mA with CuK α radiation of 1.54 Å in the 2θ range of 20–80°. The average size of metallic nanoparticles was obtained by using Debye-Scherrer equation:

1 $$ D=\frac{0.9\uplambda}{\beta \cos \uptheta} $$

where *D* is the crystallite size in nm, *λ* is the wavelength of CuK α radiation in nm, *β* is the full width at half maximum (FWHM) in radians, and *θ* is the half of the Bragg angle in radians.

Hydrodynamic diameter and polydispersity index (PDI) value of nanoparticles were determined by a DLS technique at 25 °C. Particle size analyzer (DLS-Photal, Otsuka Electronics, Japan) was employed to monitor the size distribution profile of nanoparticles with respect to intensity, number, and volume. A dispersive medium of pure water with a refractive index of 1.3328, viscosity of 0.8878, and dielectric constant of 78.3 was used as reference.

Lastly, FTIR analysis of DH-AgNps and DH-AuNps was conducted using a PerkinElmer Spectrum One FTIR spectrometer to study the interactions between the functional groups present as a source of reducing agents in the nanoparticles. The washed nanoparticle was air-dried and then analyzed by scanning the spectrum in the range of 4000–450 cm^−1^ at a resolution of 4 cm^−1^.

The stability of DH-AgNps and DH-AuNps was observed by storing the nanoparticle suspension in distilled water for different time intervals at room temperature [17]. Stability was achieved if there was no significant variation in the absorbance by UV-Vis spectrophotometer.

### Antioxidant Activity of DH-AgNps and DH-AuNps

Antioxidant activity of DH-AgNps and DH-AuNps was evaluated in vitro against free DPPH radicals according to a previous method by Brand-Williams et al. [18]. Samples were analysed spectrophotometrically at 517 nm using a UV-Vis spectrophotometer. Different concentrations (100-1000 μg/mL) of nanoparticles were assessed. The reaction mixtures were sonicated in the dark for 30 min. The radical scavenging activity was expressed as percentage inhibition:

2 $$ \left[\left({A}_{control}\hbox{--} {A}_{sample}\right)/{A}_{control}\right] \times 100 $$

where *A*_*sample*_ is the absorbance of the DPPH solution with nanoparticles and *A*_*control*_ is the absorbance of the DPPH solution without nanoparticles. Vitamin C was used as positive control.

### Antimicrobial Activity of DH-AgNps and DH-AuNps

The antimicrobial activity of DH-AgNps and DH-AuNps was evaluated against *E. coli* [ATCC 10798] and *S. aureus* [ATCC 6538]. DH-AgNps were then tested against additional pathogenic strains *P. aeruginosa* [ATCC 27853] and *S. enterica* [ATCC 13076] on Muller-Hinton agar plates. A disc-diffusion susceptibility method with slight modifications was employed for the in vitro evaluation of antimicrobial activity. An overnight log-culture of pathogenic microorganisms in lysogeny broth was diluted to reach an OD of 0.1 and spread evenly on MHA plates. Commercial-grade Neomycin (30 μg/disc) discs were maintained as positive control. Then, 30 μL of purified nanoparticle suspension containing different concentrations (i.e. 500, 1000, and 1500 μg/mL) was added onto sterile paper discs and kept for incubated at 37 °C for 24 h. After incubation, the zones of inhibition were measured [17].

### MTT Cell Proliferation Assay

RAW264.7 (Korean Cell Line Bank, Seoul, Korea) cell line was cultured in Dulbecco’s Modified Eagles medium (Gibco-BRL, Grand Island, NY, USA) supplemented with 10% fetal bovine serum and 1% penicillin/streptomycin (WelGENE Inc., Daegu, Korea) at 37 °C in a humidified atmosphere containing 5% CO_2_ and 95% air. The cytotoxicity of DH-AuNps and DH-AgNps in RAW264.7 cells were examined using 3-(4,5-dimethyl-2-thiazolyl)-2,5-diphenyl-2H tetrazolium bromide (MTT) (Life Technologies, Eugene, Oregon, USA) assay. Cells were seeded at a density of 1 × 10^4^ per well in a 96-well plate (Corning Costar, Lowell, NY, USA) and then treated with different concentrations of DH-AuNps and DH-AgNps (0–100 μg/mL) at 37 °C for 48 h at 90% confluency. After 2 days of incubation, 10 μL of MTT assay solution (5 mg/mL phosphate buffer saline) was added to each well and further incubated at 37 °C for 4 h. Then, 100 μL of dimethyl sulfoxide was added to dissolve the formazan crystals. Finally, the absorbance of each well was measured at 570 nm using an enzyme-linked immunosorbent assay reader (Bio-Tek Instruments, Inc., Vinooski, VT, USA). The optical density of formazan formed in untreated cells (negative control) represents 100% cell viability. Similarly, the cytotoxicity of nanoparticles in LPS (1 μg/ml)-induced RAW264.7 cells were conducted as previously described. [19].

### Statistical Analysis

All experiments were performed in triplicates and means with standard errors were calculated. The statistical significance of differences between values of the treated and untreated (control) groups was evaluated by one-way ANOVA. Differences with *P* < 0.05 were considered significant.

## Results and Discussion

### Biosynthesis of DH-AgNps and DH-AuNps

**Fig. 1:**
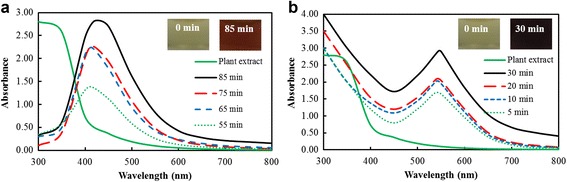
Time-dependent UV-Vis spectra of DH-AgNps (**a**) and DH-AuNps (**b**) with *A pubescens* root extract. Plant extract does not contribute to the peaks in the region of DH-AgNps and DH-AuNps. The upper right insets show that the resulting colloid suspensions are brown for DH-AgNps (**a**) and purple for DH-AuNps (**b**)

The bioreduction of silver and gold salts (1 mM) by Angelicae Pubescentis Radix extract (50%, v/v) at 80 °C was assessed by UV-Vis spectrophotometer in a time-dependent manner as observed in Fig. 1a, b. The UV-Vis spectra showed major absorption peaks at ~414 nm after 50 min for DH-AgNps and ~540 nm after 5 min for DH-AuNps. The maximum absorption values at these wavelengths (λ_max_) were obtained after 85 and 30 min of incubation for DH-AgNps and DH-AuNps, respectively. No higher absorption peaks were observed in the UV-Vis spectra recorded after these incubation periods. Similar *λ*_max_ of AgNps and AuNps have been previously reported by Singh et al. and Wang et al. [8, 20]. The synthesis was conjointly monitored visually through a color change in the reaction mixtures: insets from Fig*.* 1a, b demonstrate a color conversion of colloidal silver and gold to brown and purple, respectively due to the surface plasmon resonance phenomena: the light ray which diffuses around the colloidal nanoparticles excites the free electrons resulting in oscillations that reverberate with the frequency of visible light wavelengths [21, 22]. In addition, the UV-Vis spectrum of Angelicae Pubescentis Radix extract (50%, v/v) confirms that the extract does not contribute to the peaks observed in DH-AgNps and DH-AuNps (Fig. 1a, b).

**Fig. 2:**
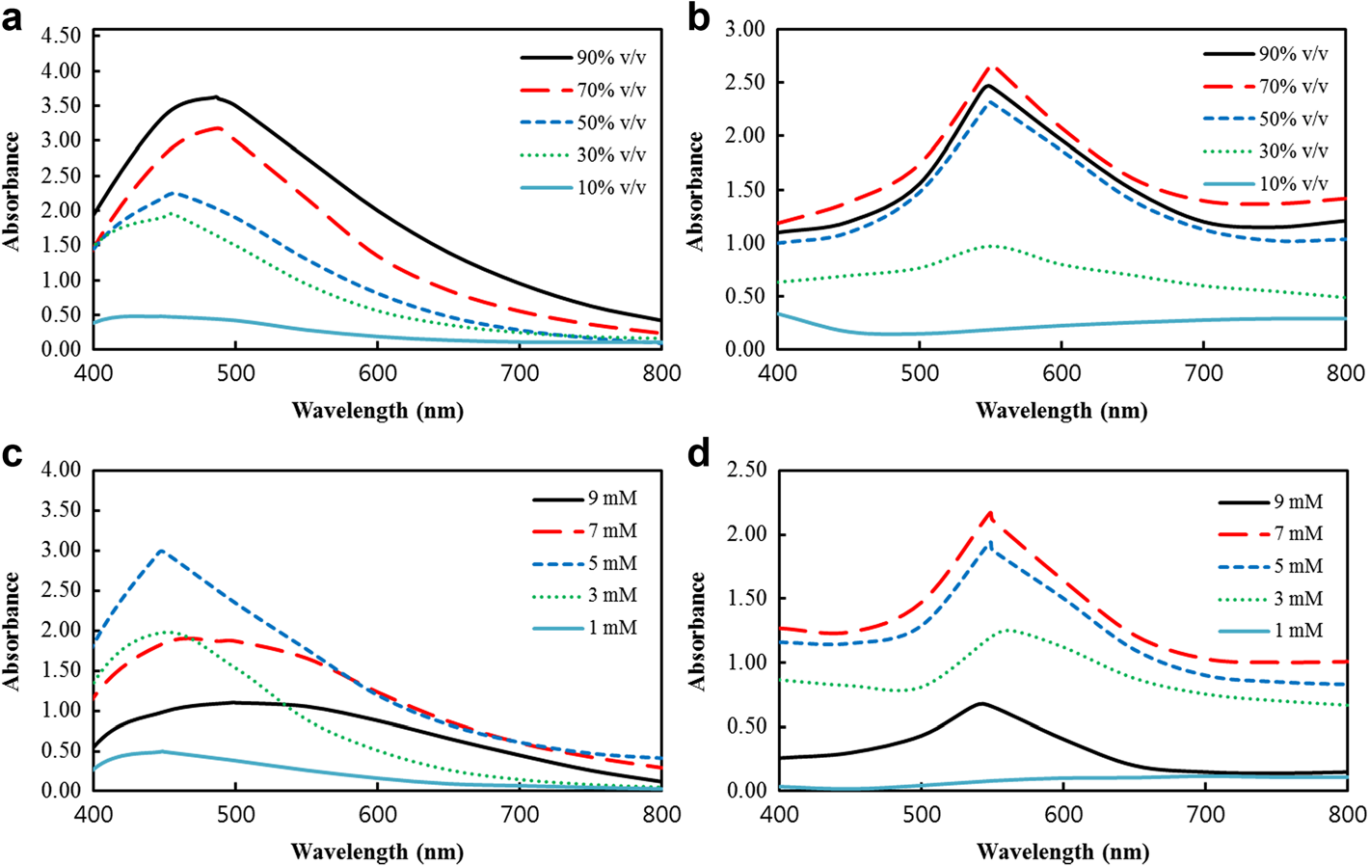
UV-Vis absorption spectra of DH-AgNps (**a**) and DH-AuNps (**b**) showing the effect of varying plant extract concentration. UV-Vis absorption spectra of DH-AgNps (**c**) and DH-AuNps (**d**) showing the effect of varying salt concentrations

The UV-Vis spectra for DH-AgNps and DH-AuNps of varying root extract concentration are given in Fig. 2a, b, respectively. Nanoparticles were synthesized with 5 mM salt concentration and incubated at 80 °C. Readings were recorded after 50 and 10 min incubation for DH-AgNps and DH-AuNps, respectively. The highest peak for DH-AgNps was obtained at root extract concentration of 90% (v/v) albeit with an induced broadening of the spectrum and a larger *λ*_max_ value. This *λ*_max_ shift to a larger value and the excessive broadening can be attributed to the increased crystallite size of the nanoparticles due to particle aggregation which decreases the sensitivity to plasmon response [5]. The sharpest peak of DH-AgNps without λ_max_ shift was obtained at 50% (v/v) root extract concentration. On the other hands, the best peak for DH-AuNps was obtained at 70% (v/v) root extract concentration. A high absorption value and sharpness of the plasmon resonance band results in a better sensing resolution for bio-imaging applications.

Figure 2c, d show the effect of silver and gold salt concentration on the synthesis of nanoparticles at 50% (v/v) root extract concentration and 80 °C. The sharpest and best peaks for DH-AgNps and DH-AuNps were obtained with 5 and 7 mM salt concentration, respectively. Increasing the concentration of silver and gold salt to 9 mM appears to enhance the broadening of the absorption curve. In addition, the intensity of the absorbance decreases, making the curve almost flat in case of DH-AgNps.

**Fig. 3:**
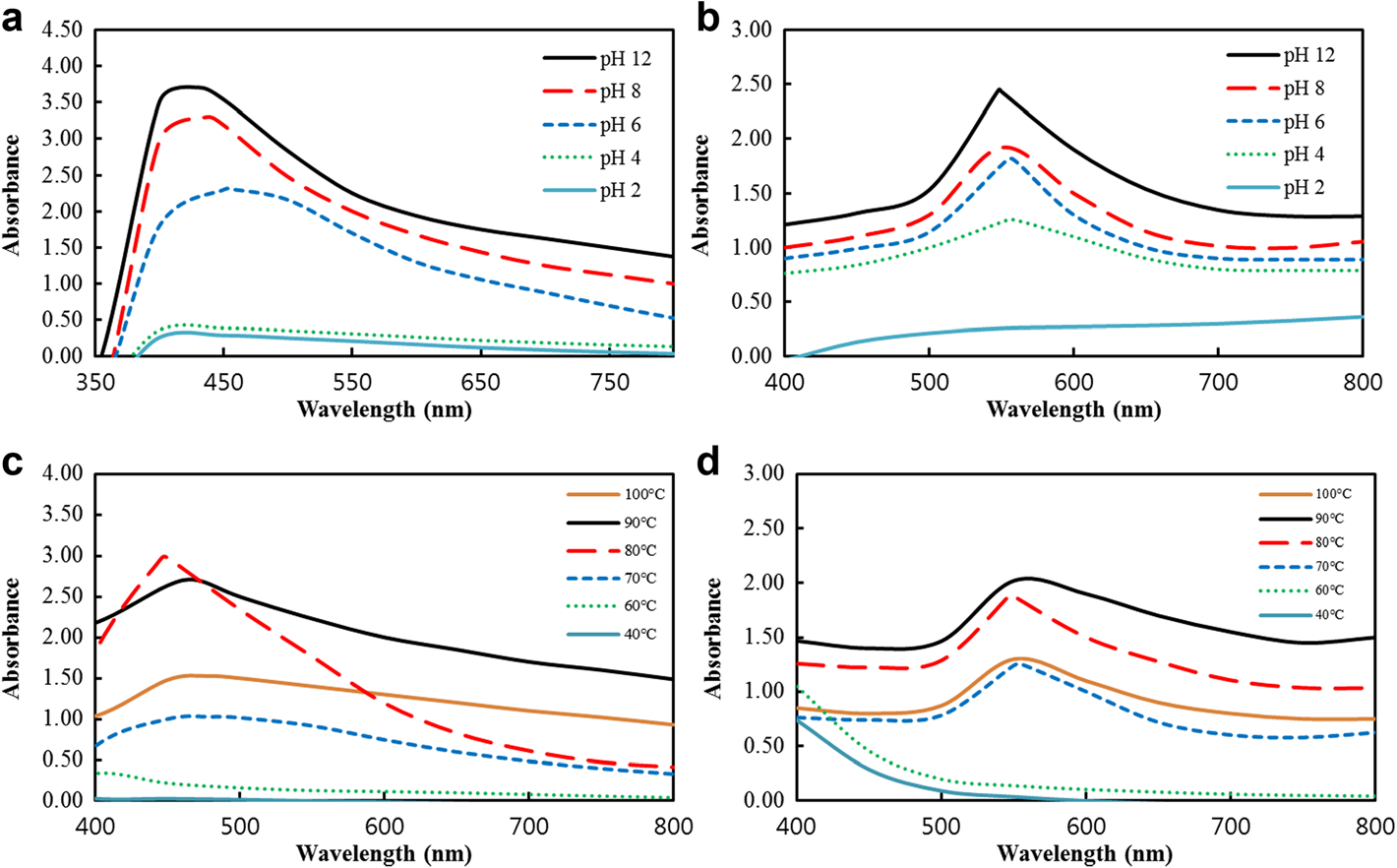
UV-Vis absorption spectra of DH-AgNps (**a**) and DH-AuNps (**b**) showing the effect of varying pH. UV-Vis absorption spectra of DH-AgNps (**c**) and DH-AuNps (**d**) showing the effect of varying temperature

Figure 3a, b show the effect of pH on the synthesis of nanoparticles. Similarly, nanoparticles were synthesized at 50% (v/v) root extract concentration and 80 °C with 5 mM salt concentration. From both UV-Vis spectra, it was observed that the highest absorption values for DH-AgNps and DH-AuNps were obtained at a basic condition; as the pH increases, an increase in the peak intensity of the UV-Vis spectra was observed for both nanoparticles. There is no shift in the *λ*_max_ associated with the changes of pH. However, in basic medium agglomeration of nanoparticles was visually detected. The nanoparticles were unstable in basic medium and exhibited aggregation which increased their particle size. This result of pH-induced nanoparticle aggregation in basic medium was also reported by Islam et al. [23]. Stability of DH-AgNps and DH-AuNps was achieved in the pH range of 2–6 where no aggregation occurred.

The biosynthesis of DH-AgNps and DH-AuNps was also subjected to varying temperature with 50% (v/v) root extract and 5 mM salt concentration (Fig. 3d, e). Increasing the temperature from 40-80 °C increases the absorption intensity. However, heating at high temperatures (90–100 °C) decreases the absorption intensity. It is speculated that high temperatures may cause the degradation of plant metabolites and aggregation of nanoparticles; the latter is especially true due to the peak broadening of the UV-Vis spectra of DH-AgNps and DH-AuNps observed in Fig. 3c, d, respectively. The sharpest peaks for DH-AgNps and DH-AuNps were obtained at 80 °C.

Lastly, the stability of the biosynthesized nanoparticles was investigated in room temperature for 7 days; similar absorption values of the reaction mixtures were observed by UV-Vis spectrophotometer at different time intervals, signifying the long-term stability of DH-AgNps and DH-AuNps.

### Characterization of DH-AgNps and DH-AuNps

**Fig. 4:**
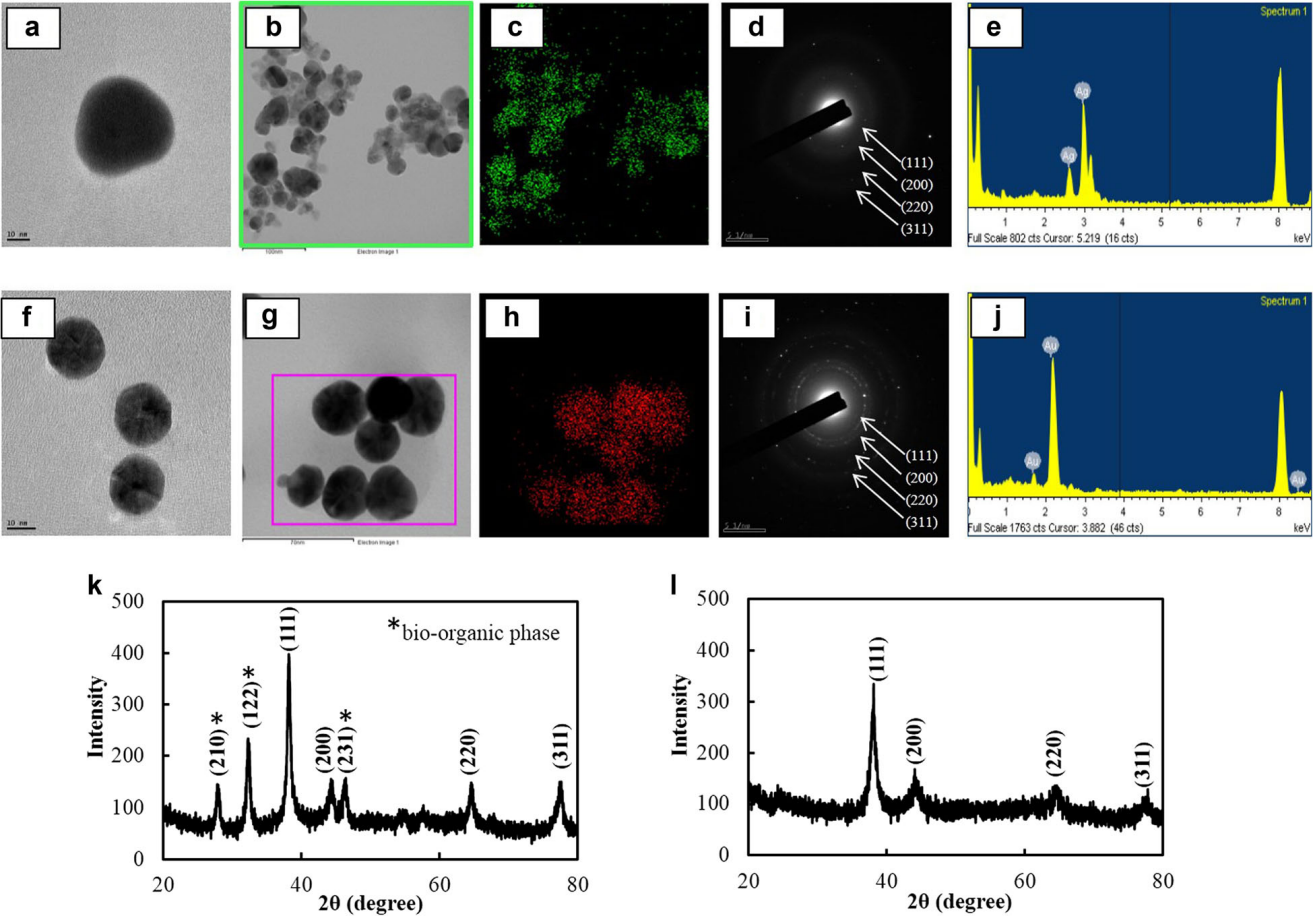
FE-TEM images of DH-AgNps (**a**) and DH-AuNps (**f**). Elemental distribution of DH-AgNps (**b**, **c**) and DH-AuNps (**g**, **h**). SAED patterns of DH-AgNps (**d**) and DH-AuNps (**i**). EDX spectrum of DH-AgNps (**e**) and DH-AuNps (**j**). XRD spectrum of DH-AgNps (**k**) and DH-AuNps (**l**)

FE-TEM images showed quasi-spherical DH-AgNps of varying sizes 20–50 nm (Fig. 4a, b) and spherical icosahedral DH-AuNps of sizes 10–30 nm (Fig. 4f, g). Elemental mapping results showed the distribution of silver (Fig. 4c) and gold (Fig. 4h) in the isolated nanoparticles. The elemental distributions of silver and gold were evidently discernible from Fig. 4b, g; silver and gold were found to be the dominant elements in the nanoparticles. EDX spectra (Fig. 4e, j) demonstrated highest optical absorption peaks at 3 and 2. 3 keV, which correspond to the characteristic peaks of silver and gold, respectively. The peaks recorded at 8 keV correspond to the copper grid used for analysis.

Characteristic peaks of XRD spectra (Fig. 4k, l) were indexed to lattice planes of Bragg’s reflection: diffraction peaks of (111), (200), (220), and (311) planes suggested that DH-AgNps and DH-AuNps were face-centered cubic and primarily composed of (111) orientation [4, 24]. The assigned peaks denoted by * at 27.89°, 32.33°, and 46.33° (2θ) (Fig. 4k) correspond to the formation of bio-organic phases of polycrystalline DH-AgNps [25, 26]. The average diameter of nanoparticles was estimated by Debye-Scherrer equation: DH-AgNps and DH-AuNps maintained average crystallite sizes of 12.48 and 7.44 nm, respectively. The electron diffraction (SAED) patterns likewise confirmed the polycrystalline nature of the nanoparticles (Fig. 4d, i).

**Fig. 5:**
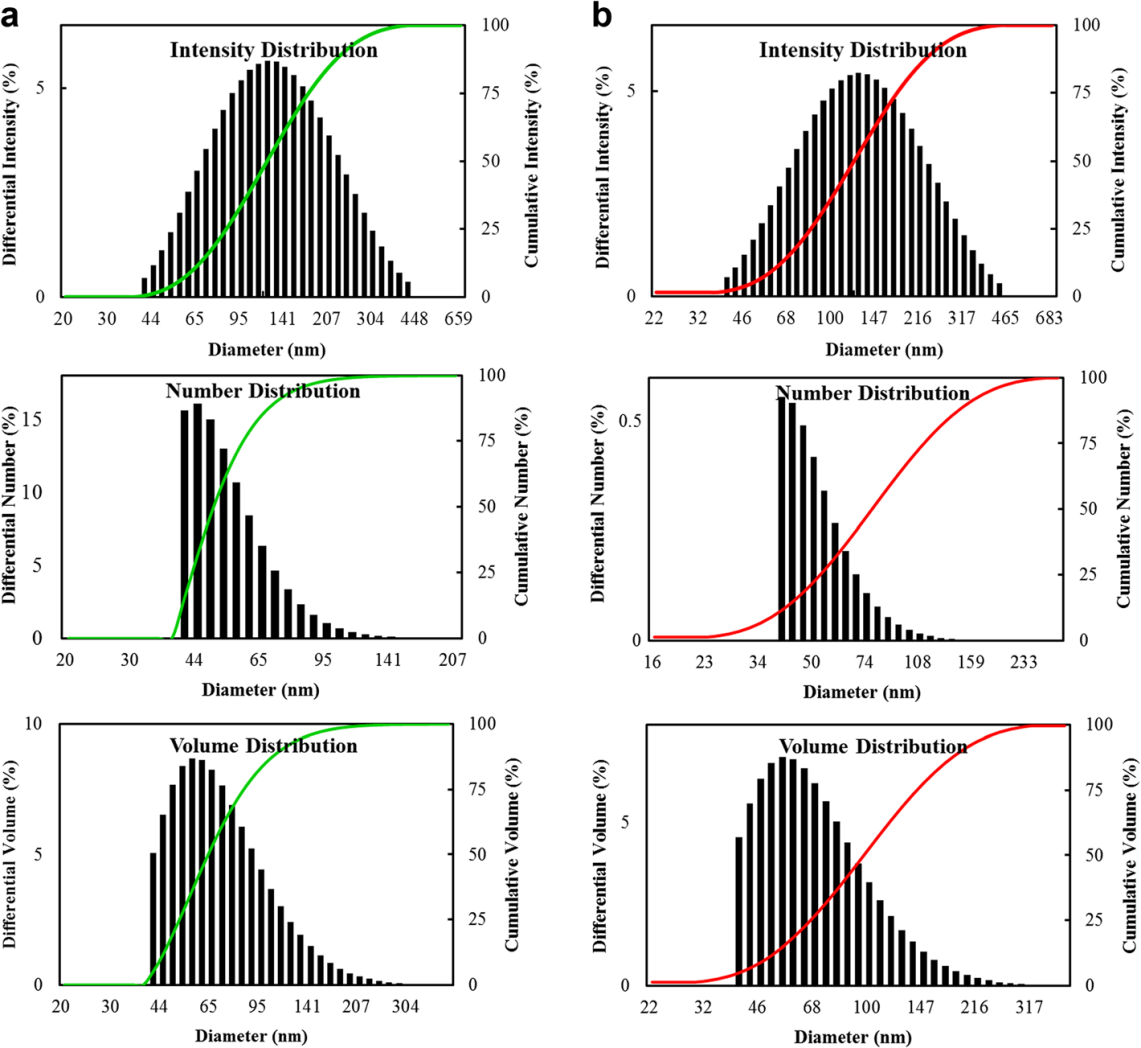
Particle size distributions of DH-AgNps (**a**) and DH-AuNps (**b**) with respect to intensity, number, and volume

The size distribution profiles of DH-AgNps and DH-AuNps by DLS method was performed using a particle size analyzer with respect to intensity, number, and volume. DLS analysis unveiled a wide range of DH-AgNps (Fig. 5a) with a Z-average value of 118 nm and a PDI of 0.23 and DH-AuNps (Fig. 5b) with a Z-average value of 109 nm and a PDI of 0.25. According to the PDI values, DH-AgNps and DH-AuNps were moderately poly-disperse. The primary and secondary metabolites of *A. pubescens* Maxim, such as coumarins, sesquiterpenes, phenols, and flavonoids, could possibly form a protective capping layer around the metallic nanoparticles which prevented the agglomeration of the nanoparticles [12, 27]. The thickness of the capping layer accounts for the discrepancy in the nanoparticle sizes analyzed by XRD and DLS since DLS method measures the hydrodynamic size of nanoparticles in aqueous suspension, which includes the metallic core and any biological molecules adhered on the particle surface [28].

**Fig. 6:**
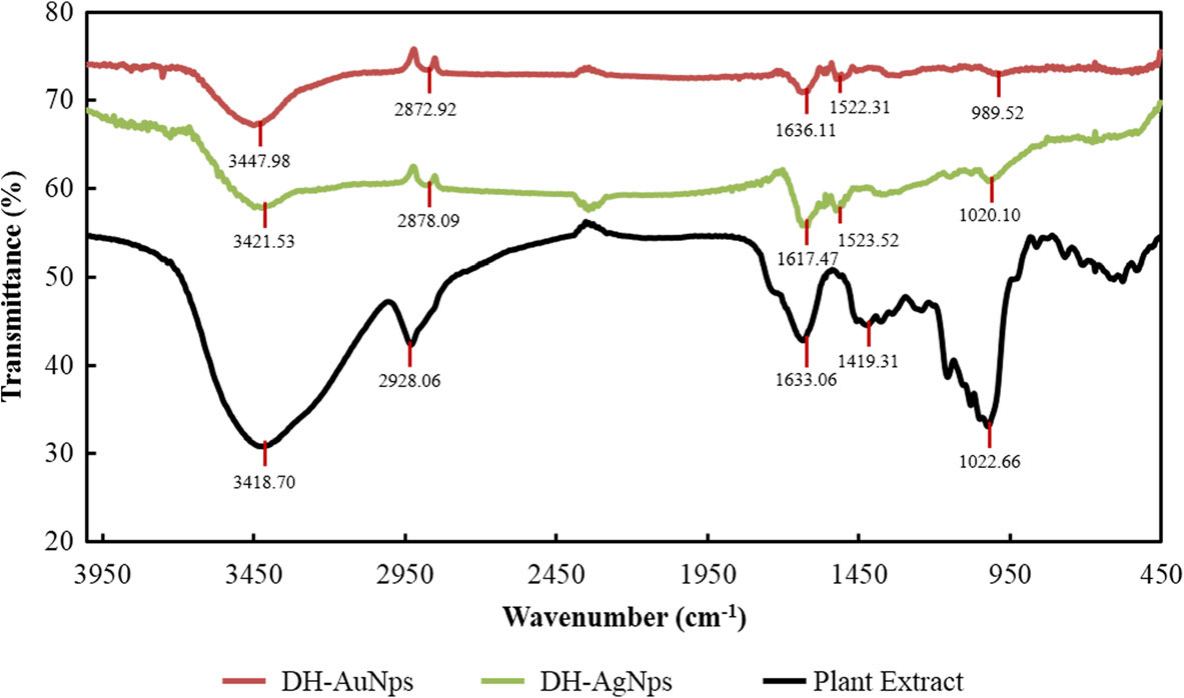
FTIR spectra of biosynthesized nanoparticles and plant extract

FTIR spectra of DH-AgNps (green) and DH-AuNps (red) were compared against the spectrum of aqueous extract of Du Huo (black, positive control) (Fig. 6). It was apparent from Fig. 6 that DH-AgNps and DH-AuNps acquired additional bands which originated from plant extract. FTIR spectrum of DH-AgNps showed bands at 3421.53 and 2878.09 cm^−1^ corresponding to the stretching of O-H bond of alcohol groups and C-H bond of alkanes. The presence of these bands suggests the roles of flavonoids and sesquiterpenes in the capping layer of the nanoparticles, respectively. Additionally, characteristic peaks at 1617.47–1523.52 cm^−1^ correspond to aromatic C=C bond stretching which is due to the phenolic compounds on the surface of the nanoparticles. Lastly, the intense band located at 1020.10 cm^−1^ is affiliated to the ether groups (C–O bond stretch) [10, 29]. Similar characteristic peaks were also observed in the FTIR spectrum of DH-AuNps. Major characteristic peaks of flavonoids, sesquiterpenes, and phenols suggest that these biomolecules may be responsible for the formation of protective capping layers around DH-AgNps and DH-AuNps which grant protection against aggregation and biological activity. Flavonoids, terpenes, and phenols are strong reducing agents which may reduce silver and gold salts into their respective nanoparticles and attach on the nanoparticle surface, thereby providing stabilization through electrostatic interaction [4].

### Antioxidant Activity of DH-AgNps and DH-AuNps

**Fig. 7:**
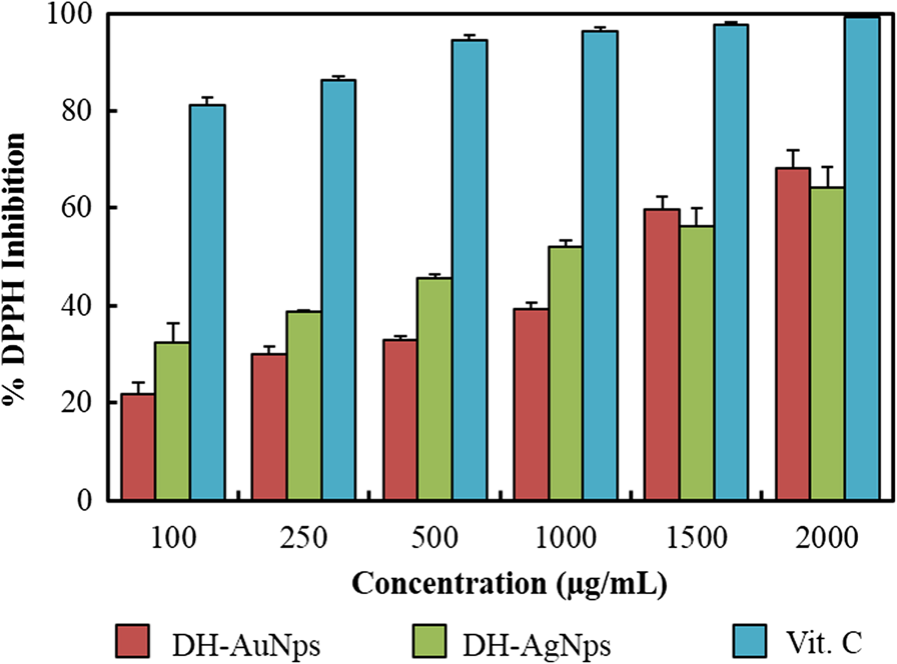
Dose-dependent in vitro DPPH radical scavenging activity of biosynthesized nanoparticles

The antioxidant activity of DH-AgNps and DH-AuNps was evaluated against DPPH free radicals as shown in Fig. 7. DPPH consists stable free radical molecules and is readily reduced by accepting hydrogen or electron from nanoparticles [30]. Figure 7 shows the dose-dependent scavenging activity of DH-AgNps and DH-AuNps. The IC_50_ values were determined by linear regression and were calculated to be 1.01 and 1.23 mg/mL for DH-AgNps and DH-AuNps, respectively. The antioxidant activity of biosynthesized nanoparticles can be attributed to the antioxidant property of Angelicae Pubescentis Radix [15]. These results suggest that the protective capping layer of DH-AgNps and DH-AuNps by flavonoids, sesquiterpenes, and phenols seems to be the major contributors to the free radical scavenging activity. The antioxidant activities of biosynthetic AgNps and AuNps by aqueous extract of *Solanum torvum* fruit, *Cassia tora* leaves, and *Atrocarpus altilis* leaves have been reported [30–32]. This is the first study to report the antioxidant activity of AgNps and AuNps synthesized by the aqueous extract of *A. pubescens* Maxim. This green synthesis is economical, eco-friendly, and conducive for the development of cheaper and newer antioxidant agents in biomedicine.

### Antimicrobial Activity of DH-AgNps

**Fig. 8:**
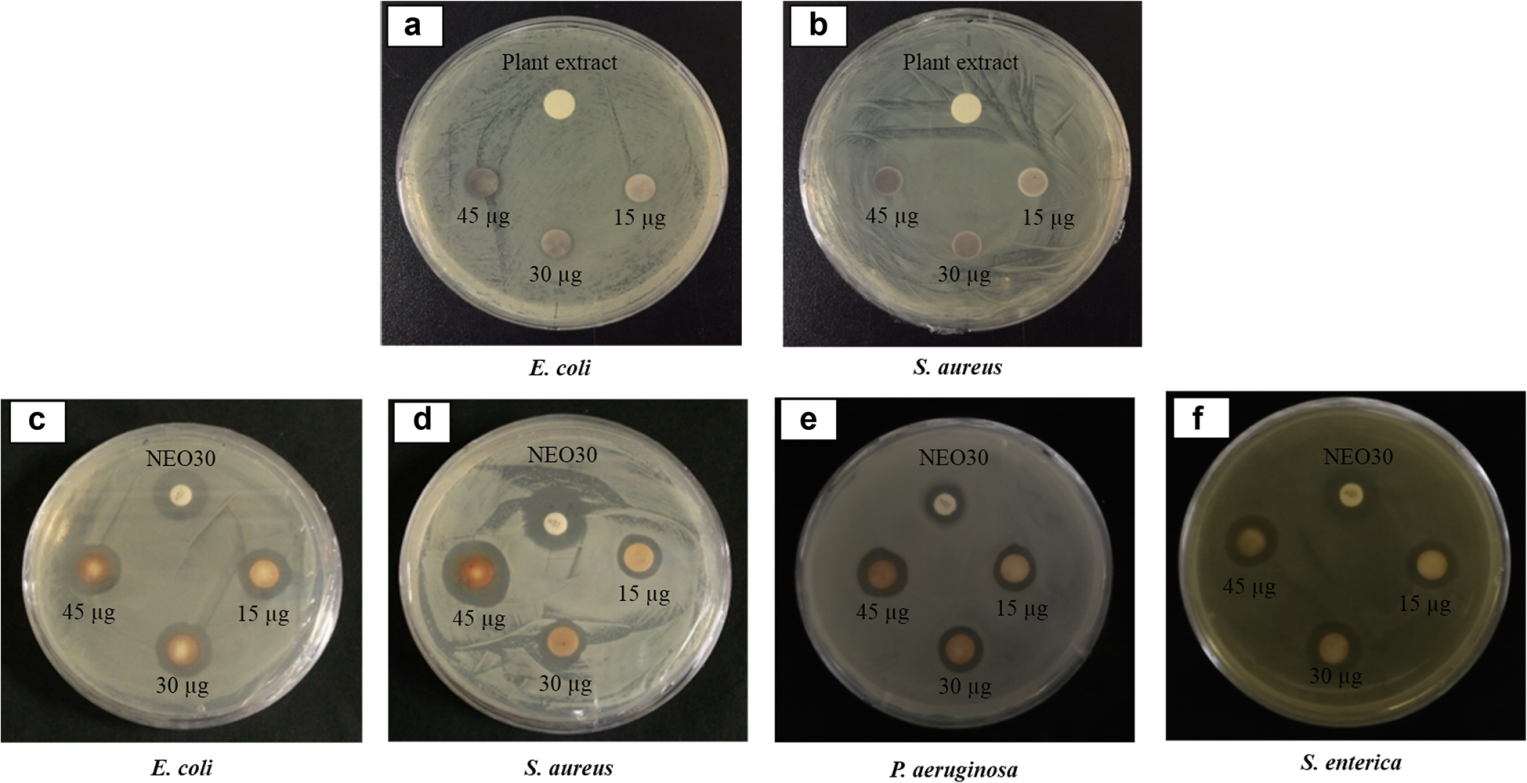
Zones of inhibition of purified DH-AuNps suspensions (30 μL) and plant extract against *E. coli* (**a**) and *S. aureus* (**b**). Zones of inhibition of purified DH-AgNps suspensions (30 μL) and Neomycin (NEO30) as standard antibiotics as control against *E. coli* (**c**), *S. saureus* (**d**), *P. aeruginosa* (**e**), and *S. enterica* (**f**)

The antimicrobial activity of DH-AgNps, DH-AuNps, and *A. pubescens* root extract (50%, v/v; 30 μL) was examined by disc-diffusion susceptibility method against *E. coli* and *S. aureus* as shown in Fig. 8a, b. No zones of inhibition were observed in Fig. 8a, b, meaning that DH-AuNps and root extract do not possess antimicrobial activity against the Gram negative and Gram positive representative bacteria. On the other hand, zones of inhibition of DH-AgNps began to form around paper discs after 24 h incubation at 37 °C (Fig. 8c, d). To evaluate the effectiveness of DH-AgNps compared to Neomycin (30 μg/disc), 30 μL of purified DH-AgNps suspension containing different concentrations was added onto sterile paper discs to generate 15, 30, and 45 μg/disc. The mean diameters of the zones in triplicates were measured and interpreted in Table 1.

**Table 1:** Diameter of zone of inhibition (mm) of DH-AgNps against pathogenic microorganisms

Pathogenic microorganisms	Zone of inhibition (mm)^a^			
	NEO30 (30 μg/disc)	15 μg/disc	30 μg/disc	45 μg/disc
*Escherichia coli* [ATCC 10798]	15.20 ± 0.20	12.75 ± 0.85	13.34 ± 0.88	14.00 ± 1.15
*Staphylococcus aureus* [ATCC 6538]	15.60 ± 0.40	15.20 ± 0.58	16.00 ± 0.55	17.30 ± 0.44
*Pseudomonas aeruginosa* [ATCC 27853]	11.50 ± 0.50	12.00 ± 0.00	12.25 ± 0.25	13.00 ± 0.50
*Salmonella enterica* [ATCC 13076]	15.00 ± 0.00	11.25 ± 2.75	14.00 ± 1.00	12.00 ± 2.00

^a^Mean diameter of three discs; 30 μL of suspensions containing purified nanoparticles

The results clearly demonstrated that DH-AgNps exerted visible zones of inhibition against the pathogenic models of Gram-positive and Gram-negative bacteria. Furthermore, the antimicrobial activity of DH-AgNps was tested on other pathogenic microorganisms, including *P. aeruginosa* and *S. enterica* (Fig. 8e,f). Based on the zones of inhibition, DH-AgNps exhibited a maximum dose-dependent antimicrobial activity against *S.aureus* followed by *E. coli*, *P. aeruginosa*, and finally *S. enterica*. Interestingly, the antimicrobial effect of DH-AgNps against *S.aureus* (16.00 ± 0.55 mm) and *P. aeruginosa* (12.25 ± 0.25 mm) was determined to be slightly better than that of commercial antibiotic disc (15.60 ± 0.40 mm; 11.50 ± 0.50 mm) with the same concentrations of DH-AgNps (30 μg/disc) and antibiotics (30 μg/disc). These results indicated that DH-AgNps were able to effectively diffuse through paper discs and inhibited the growth of the tested pathogenic microorganisms. Many studies have reported the antimicrobial activity of AgNps against pathogenic microorganisms [1]. Intriguingly, AgNps may also enhance the antimicrobial activity of antibiotics against resistant bacteria through a synergistic mechanism [33]. Nevertheless, the exact mechanism of AgNps has yet to be illuminated. However, it was proposed that the accumulation of positively-charged silver ions released by AgNps on the bacterial membrane caused a charge influx, resulting in the increased cell permeability of silver ions into the cells and ultimately cell death [34]. Additionally, free silver ions can bind with the thiol (S-H) groups in enzymes and proteins to inhibit bacterial oxygen metabolism (i.e., cellular respiration) [35]. In short, the biosynthesized DH-AgNps may be found useful as green antimicrobial alternatives in clinical settings against pathogenic microorganisms with increased efficacy compared to commercial antibiotic.

### Cytotoxicity Evaluation of DH-AgNps and DH-AuNps in RAW264.7 Cells

**Fig. 9:**
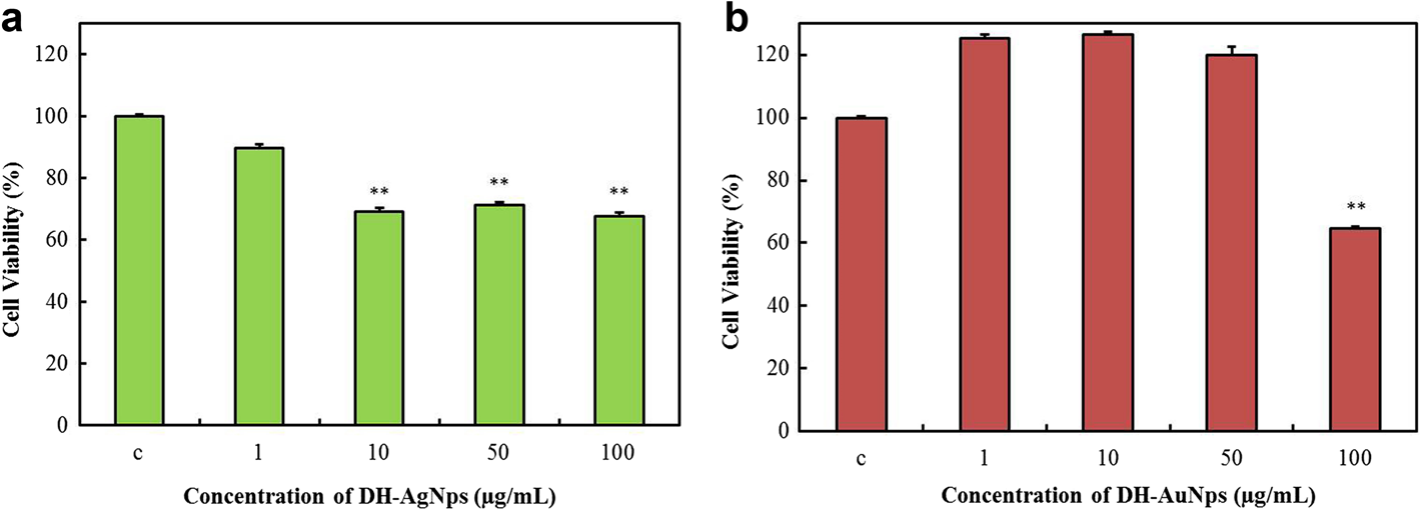
Dose-dependent cytotoxicity of DH-AgNps (**a**) and DH-AuNps (**b**) after 48 h of treatment in murine macrophage (RAW264.7). Notes: **P < 0.05 versus control (untreated group). The statistical significance of differences between values was evaluated by one-way ANOVA. Abbreviation: c, control (untreated group)

In this current study, RAW264.7 and LPS-induced RAW264.7 cells served as models to determine the cytotoxicity of DH-AgNps and DH-AuNps in non-diseased and diseased condition. LPS was used to induce strong inflammatory response in normal mammalian cells. In vitro cytotoxicity assay of DH-AgNps and DH-AuNps was performed by MTT. Both nanoparticles were evaluated at concentrations ranging from 1 to 100 μg/mL. DH-AgNps showed cytotoxicity toward RAW264.7 cells at a concentration ≥10 μg/mL with over than 30% cell death at a treatment concentration of 100 μg/mL (Fig. 9a). However, at a concentration ≤1 μg/mL, no significant cytotoxicity was observed, implying its safe usage at this concentration. On the other hand, when exposed to concentrations of 1–50 μg/mL of DH-AuNps, RAW264.7 cell lines did not exhibit cell death and continued to proliferate (Fig. 9b). Cell inhibition started to become more significant at 100 μg/mL. These results indicate that DH-AuNps may not exhibit cytotoxicity to normal cells at this condition.

**Fig. 10:**
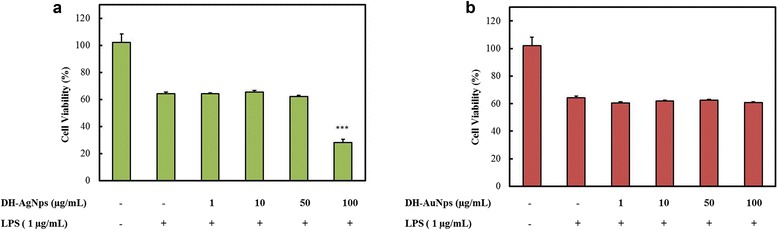
Dose-dependent cytotoxicity of DH-AgNps (**a**) and DH-AuNps (**b**) after 48 h of treatment in LPS-stimulated murine macrophage (RAW264.7). Notes: ***P < 0.001 versus group treated with LPS alone. The statistical significance of differences between values was evaluated by one-way ANOVA

For evaluating the cytotoxicity in diseased condition, LPS-stimulated RAW264.7 cells were used. The same concentrations of DH-AgNps and DH-AuNps were employed as shown in Fig. 10a, b. Based on these results, the plant-mediated nanoparticles did not worsen the already existing cytotoxicity caused by LPS stimulation after 48 h of treatment; however, 100 μg/mL of DH-AgNPs showed reduction in growth of cells. Due to the non-cytotoxity of DH-AuNps in RAW264.7 and LPS-induced RAW264.7 cells, DH-AuNps may be considered for potential biomedical applications in drug delivery and molecular imaging to sites of inflammation without causing irritation onto the afflicted cells.

## Conclusions

The root extract of Angelicae Pubescentis Radix acts as reducing and stabilizing agents for the straightforward and facile synthesis of DH-AgNps and DH-AuNps without the use of hazardous chemicals. The optimal root extract and metal ions concentrations for DH-AgNps are 50% (v/v) and 5 mM; the optimal root extract and metal ions concentrations for DH-AuNps are 70% (v/v) and 7 mM. The nanoparticles were unstable and exhibited aggregation in basic medium (pH 8-12). The optimal temperature for DH-AgNps and DH-AuNps without the broadening of peak spectra is 80 °C. The biosynthesized nanoparticles were extensively characterized by UV-Vis spectroscopy, FE-TEM, EDX, elemental mapping, XRD, SAED, DLS, and FTIR spectroscopy. The biosynthesized AgNps and AuNps were stable for 7 days at room temperature and demonstrated potentials as novel antioxidant agents while the latter showed antimicrobial effect against pathogenic *E. coli*, *S. aureus*, *P. aeruginosa*, and *S. enterica*. The cytotoxicity of DH-AgNps and DH-AuNps was evaluated in RAW264.7 and LPS-stimulated RAW264.7 cell line: DH-AuNps demonstrated favorable applications as drug delivery agents to sites of inflammation attributed to their non-cytotoxicity to normal and diseased cells.

## Abbreviations

AgNps: Silver nanoparticles
AuNps: Gold nanoparticles
DH-AgNps: Silver nanoparticles mediated by Du Huo
DH-AuNps: Gold nanoparticles mediated by Du Huo
DLS: Dynamic light scattering
DPPH: 2,2-diphenyl-1-picrylhydrzyl
EDX: Energy-dispersive X-ray
FE-TEM: Field emission-transmission electron microscopy
FTIR: Fourier transform infrared spectroscopy
FWHM: Full-width at half maximum
LPS: Lipopolysaccharide
MTT: 3-(4,5-dimethyl-2-thiazolyl)-2,5-diphenyl-2H tetrazolium bromide
PDI: Polydispersity index
SAED: Selected area electron diffraction
UV-Vis: Ultraviolet-visible
XRD: X-ray powder diffraction
